# The Environmental Price Tag on a Ton of Mountaintop Removal Coal

**DOI:** 10.1371/journal.pone.0073203

**Published:** 2013-09-11

**Authors:** Brian D. Lutz, Emily S. Bernhardt, William H. Schlesinger

**Affiliations:** 1 Department of Biology, Kent State University, Kent, Ohio, United States of America; 2 Department of Biology, Duke University, Durham, North Carolina, United States of America; 3 Cary Institute of Ecosystem Studies, Millbrook, New York, United States of America; University of Florida, United States of America

## Abstract

While several thousand square kilometers of land area have been subject to surface mining in the Central Appalachians, no reliable estimate exists for how much coal is produced per unit landscape disturbance. We provide this estimate using regional satellite-derived mine delineations and historical county-level coal production data for the period 1985–2005, and further relate the aerial extent of mining disturbance to stream impairment and loss of ecosystem carbon sequestration potential. To meet current US coal demands, an area the size of Washington DC would need to be mined every 81 days. A one-year supply of coal would result in ∼2,300 km of stream impairment and a loss of ecosystem carbon sequestration capacity comparable to the global warming potential of >33,000 US homes. For the first time, the environmental impacts of surface coal mining can be directly scaled with coal production rates.

## Introduction

Mountaintop removal coal mining (MTR) is a particularly invasive mining practice developed in the United States (US) capable of producing low-cost coal. The process of MTR uses explosives and heavy machinery to remove entire mountain ridges in order to access near surface coal deposits, producing vast quantities of mine spoil that fills valleys and buries streams. MTR has expanded dramatically in recent decades and is now the dominant driver of land-use change across the Central Appalachian region [1].

Growing scientific evidence demonstrates that these surface mining activities present severe, negative environmental consequences including widespread destruction of forest habitat, long-term impairment of ecosystem carbon and nutrient cycling, and regional deterioration of stream water quality [2]. Yet, despite this, MTR remains highly controversial. This is not because the scientific evidence is equivocal, but rather because the environmental costs of these surface mining activities must be weighed against the economic benefits of coal production. Industry employment data [3], coal revenues [4], and severance taxes [5], [6] have all historically been calculated on a per-unit-coal basis, making the economic effects of policy decisions to increase or decrease production directly and transparently quantifiable. The same has not been true for the environmental costs; while there have been many assessments of MTR impacts we have yet to translate our understanding of these effects into units of coal produced [7]. It is difficult, if not impossible, for policymakers to weigh the costs and benefits of MTR if the environmental impacts are not conveyed in terms of coal production.

During the process of mining, forests are cleared and the soil and bedrock overlying coal seams (overburden) are removed. The land surface is dramatically reengineered, leaving behind a new topography constructed of reshaped mine spoil. Post-mining soil profiles often have higher bulk densities, lower water infiltration rates, and lower nutrient contents [2]. Most reclaimed mines are seeded with grasses and support little woody vegetation regrowth, even many years after site reclamation – representing a long-term loss of forest habitat [2], as well as a loss of ecosystem carbon (C) sequestration potential as forests are converted to grasslands [8], [9]. Additionally, surface coal mining in this region produces alkaline mine drainage containing high concentrations of ions and various solutes that can be harmful to aquatic biota in receiving streams [2]. Recent studies have demonstrated that all of these environmental impacts – habitat loss, reduced ecosystem C sequestration, and stream water quality impairment – are directly related to the amount of land area disturbed by mining activities [8]–[10]. Thus, in order to quantify these environmental impacts in units of coal produced, it is necessary to know how much coal is produced per unit area of mining disturbance.

While adequate data are available describing the amount of land area *permitted* for mining, not all permitted areas are ultimately mined; single mines occupy large tracts of land (up to 88 km^2^) and topographic variation makes some coal difficult to access [7]. Regulatory agencies have not recorded reliable data on the total permitted area that is ultimately mined [7], requiring that this information be gained through alternative methods. For 47 counties in southern West Virginia and eastern Kentucky – a study area occupying ∼82% of the Central Appalachian coal region (59,569 km^2^) [11] – we use estimates of surface mining disturbances derived from historical satellite imagery for the period 1985–2005 [10], and regress the aerial extent of mining disturbances against cumulative county coal production over this period. Using this newly derived estimate of coal produced per unit disturbance, we then convert previously published estimates of the environmental impacts of MTR into units of coal production. Further, we bring new perspective to the ongoing debate surrounding MTR by placing these environmental costs in terms of regional coal production rates, as well as in terms of total US coal demand.

## Methods

Mined areas were delineated at decadal intervals (1976, 1985, 1995, and 2005) using previously published methods [10]. Briefly, digital images from the Multispectral Scanner (MSS, 80 m resolution) and Thematic Mapper (TM, 30 m resolution) taken during mid-summer (in order to minimize seasonal variation in illumination and maximize contrast between disturbed areas from surrounding forests) were reviewed to ensure no interference from haze, smog or cloud cover. Historical topographic data (pre-mining) were obtained from the Defense Mapping Agency (U.S. Department of Defense; Digital Terrain Elevation Data, Level I; https://www1.nga.mil/ProductsServices). The hyperspherical direction cosine (HSDC) method [12] was used to reduce albedo-related variations in illumination, and training samples were selected for each imagery date to classify land-cover based on the Anderson Level II system [13]. Erdas IMAGINE and GIS software was used for image analysis based on a two-stage classification process: (1) pixel-based spectral signatures using the supervised maximum likelihood technique [14] were identified for different land cover types, and (2) decision tree analysis was used to classify mined areas. Mined areas were defined as any bare rock or soil land cover that was not within a 400 m buffer surrounding highways, rivers, or agricultural areas. Final products were raster datasets of 30 m resolution for each time step with binary values indicating mining disturbance or not. For more details on mine delineation methodology, see Bernhardt et al. [10].

Surface mines have long lifecycles often exceeding 10 years [7], thus decadal-scale imagery was adequate for capturing most mining activity [10]. Annual county-level coal production data were obtained for the 33 counties within the study area located in eastern Kentucky [15] and for the 14 counties in southern West Virginia [16]. While satellite imagery and mine delineations extended back to 1976, coal production data were only available for all counties beginning in 1985. Therefore, the cumulative extent of mining disturbance over the period 1985–2005 was calculated as the areas that were observed to have been disturbed by surface mining in 1995 and 2005 that were not already disturbed in 1985 or earlier.

Simple linear regression (SLR) was used to estimate the relationship between county-level coal production and aerial mining disturbance extent (*lm* function, *Stat* package, CRAN-R statistical software) [17]. One county (Pike, KY) had more than twice the mining disturbance compared to any other county (Table S1 in File S1), and including this county in the analysis substantially decreased the regression slope estimate of how much coal is typically produced per unit disturbance. Although this decrease was not statistically significant, this county was omitted from the regression analysis due to the high leverage exerted by this single data point. Because omitting this data point increases our estimate of how much coal is produced per unit area disturbance, our estimates of the environmental impacts of MTR may be slightly conservative.

In a previous study, we estimated the threshold value of watershed mining impacts at which a receiving stream is likely to be classified as biologically impaired (as defined by significant losses of pollution intolerant stream macroinvertebrates) [10]. We estimated the extent of mining within every watershed throughout 14 counties in southern West Virginia using the same satellite-derived mine delineations used here, and through multiple modeling approaches we assessed macroinvertebrate responses across sites spanning a gradient of mining intensity. Using two separate models we assessed the responses of commonly used biotic indices as a function of catchment mining intensity, the West Virginia Stream Condition Index (WVSCI) [18] and the Genus-Level Index of Most Probable Stream Status (GLIMPSS) [19]. We used a third model, Threshold Indicator Taxa Analysis (TITAN) [20], to assess the response of individual taxa along the mining gradient. These different models were highly consistent with one another, indicating significant impairment of the stream macroinvertebrate community occurs once 2.2–6.3% of the surface area in their watersheds is converted to mines (Table 1) [10]. We were unable to include data for KY in this previous study due to differences between state data availability and sampling protocols, though there are no distinguishing characteristics that would suggest macroinvertebrate communities in KY streams would respond differently to mining intensity than those in WV. In our previous analysis we estimated cumulative stream impairment at decadal intervals over the period 1976–2005; here we truncate the values to only consider the extent of stream impairment having occurred after 1985 to be temporally consistent with the available coal production data (Table 1). Over the period 1985–2005 an estimated 653 km^2^ was mined across the 14 WV counties. Based on the different thresholds for stream impairment predicted by the different models, we estimate this resulted in 1,763–1,968 km of stream channel becoming impaired, or 27.0–30.2 m of stream channel length lost for each hectare of surface coal mining disturbance (Table 1). We use the SLR slope estimate relating coal production to disturbance (tons coal ha^−1^) to translate this estimate of stream impairment (m ha^−1^) into units of coal produced (cm tons coal^−1^).

**Table 1:** Stream impairment estimates per unit coal produced.

Metric	Mining threshold (%)^a^	Cumulative impairment through 2005 (km)^b^	Impairment from 1985 and prior (km)^c^	Impairment for period 1985–2005 (km)	Stream impairment per unit disturbance (m ha^−1^)^d^	Stream impairment per unit coal (cm ton^−1^)^e^
*WVSCI*	5.4	2,834	940	1,894	29.0	0.25
*GLIMPSS*	6.3	3,390	1,422	1,968	30.2	0.26
*TITAN*	2.2	4,308	2,545	1,763	27.0	0.24

a Values correspond to upper 95% CI values reported by Bernhardt et al. (*see* Table 1 their publication) [10].

b Values from Bernhardt et al. [10] for cumulative stream impairment over the period 1976–2005.

c Data provided in Fig. 4 of ref.[10], as well as by the authors.

d Quotient of stream impairment (1985–2005) divided by mining disturbance (1985–2005 WV only  = 65,211ha; from Table S1 in File S1).

e Converted to stream impairment per unit coal using slope estimate (11,500 tons coal/ha).

Clearing of forests prior to mining can result in large initial losses of soil and biomass C pools. These ecosystems will re-accumulate C following mine reclamation, but likely at a slower rate because post-mining soils are often compacted with low nutrient content and reduced fertility [2] – all of which affect vegetation re-establishment and, thus, limit C sequestration rates. This has been especially true since the passage of the Surface Mining Control and Reclamation Act (SMCRA) of 1977, which has favored severe soil compaction and reseeding with grasses in order to reduce sediment losses from mined areas [21]. We estimate the amount of foregone C sequestration by comparing previously published data for ecosystems recovering from mining to the C sequestration potential of unmined sites. Simmons et al. [8] inventoried soil and biomass C pools 15 years after a forested site in the Appalachian coal region was cleared, mined and restored to grassland following SMCRA regulations. We divide the reported C pool values by the number of years since mining to estimate annual ecosystem C sequestration rates. Biomass and soil carbon in this system accumulated at rates of 8 and 159 g C m^−2^ yr^−1^, respectively (Table 2). Amichev et al. [9] reported ecosystem C soil and biomass pool estimates for 14 pre-SMCRA mining sites across the region exhibiting forest regrowth (“reclaimed forests”), as well as 8 non-mined reference forests (“unmined forests”). We estimate C sequestration rates for these reclaimed and unmined forests by dividing their soil and biomass pool estimates by the site-specific stand ages. The reclaimed forest sites accumulated biomass and soil carbon at 274 and 83 g C m^−2^ yr^−1^, while the unmined forest sites showed C accumulation rates of 237 and 152 g C m^−2^ yr^−1^ for biomass and soil pools (Table 2). Thus, the net ecosystem production rate (soil + biomass C accumulation) for unmined forests (389 g C m^−2^ yr^−1^) exceeds that for reclaimed mine sites converted to grasslands (167 g C m^−2^ yr^−1^) or sites supporting forest regrowth (357 g C m^−2^ yr^−1^). Our estimate of foregone ecosystem C sequestration resulting from mining, then, ranges from 32–222 g C m^−2^ yr^−1^. Because most sites mined post-SMCRA (after 1977) have been converted to grasslands [21], the amount of foregone ecosystem C sequestration for the mining disturbance that occurred over our study period is likely at the upper end of the reported range (222 g C m^−2^ yr^−1^). We divide this estimate of foregone ecosystem C sequestration per unit area disturbance by the regression value of coal produced per unit area disturbance to derive our final estimate of foregone ecosystem C sequestration in units of coal produced.

**Table 2:** Foregone ecosystem C sequestration per unit coal produced.

Study	Ecosystem Type	Soil C Sequestration Rate(g C m^−2^ yr^−1^)	Biomass C Sequestration Rate(g C m^−2^ yr^−1^)	Ecosystem C Sequestration Rate(g C m^−2^ yr^−1^)
*Simmons et al.*	Reclaimed Grassland	159^a^	8^a^	167
*Amichev et al.*	Reclaimed Forests	274^b^	83^b^	357
*Amichev et al.*	Unmined Forests	237^b^	152^b^	389

a Values are from Simmons et al. Table 5 [8]. Our reference to “Soil” is considered “total belowground” by the authors; our use of “biomass” is considered “total aboveground, as reported by the authors. Reported values for C pools were divided by stand age (15 yr) and converted to g C m^−2^ yr^−1^.

b Values are calculated from Amichev et al. Table 1 [9]. Reported values for C pools for all sites were divided by stand age and converted to gC m^−2^ yr^−1^. Our reference to “Soil” is considered “SOC” + “Litter Layer C”, as reported by the authors; our reference to “Biomass” is considered “Total Tree C”, as reported by the authors.

To bring perspective to these per-unit-coal estimates of the environmental costs of MTR, we calculate the extent of environmental impacts across the region by multiplying the per-unit-coal values by the cumulative annual coal production data across all counties. While we could also estimate the extent of these impacts directly from the amount of land area identified as having been mined in the satellite imagery analysis, the imagery analyses are only of decadal resolution while the coal production data are reported annually. Thus, using the coal production data allow us to represent the expansion of these environmental impacts with higher temporal resolution.

## Results and Discussion

**Figure 1:**
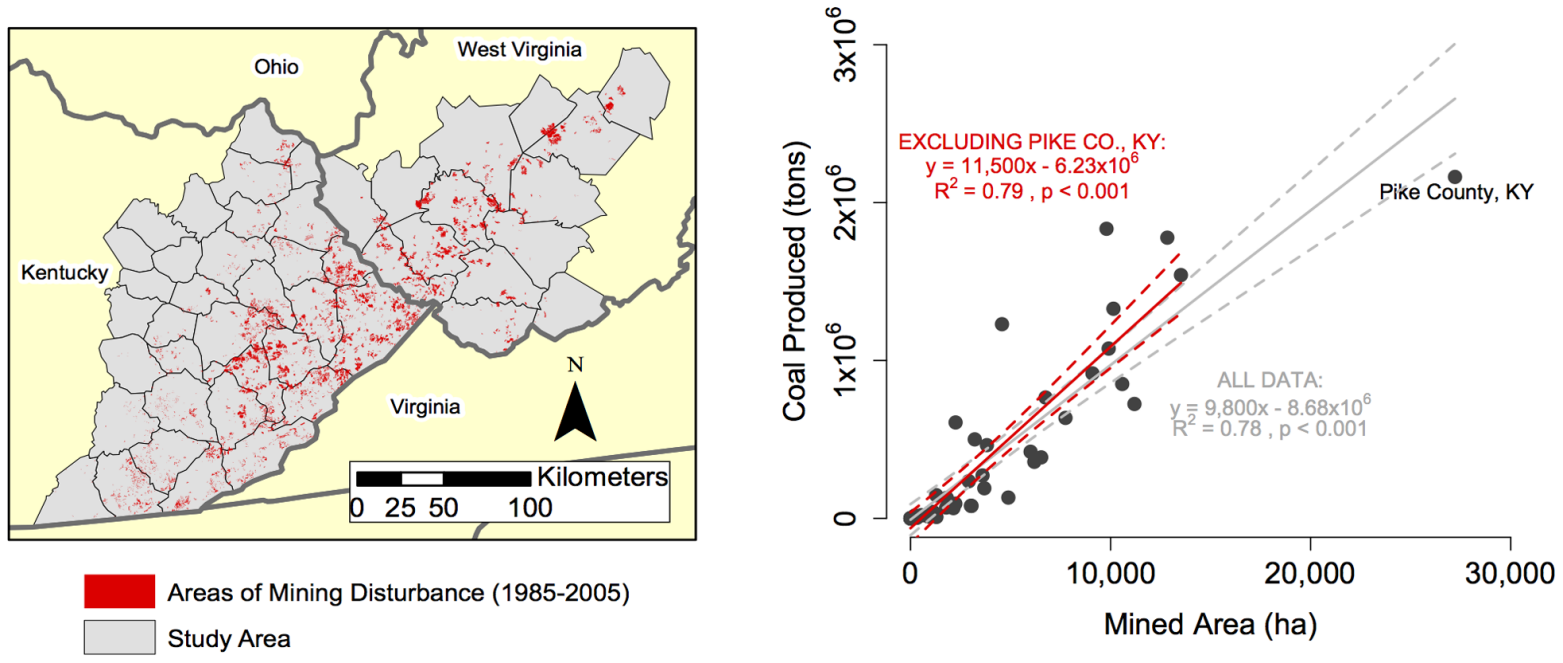
Extent of mining disturbance and relationship to coal production. Red areas show the cumulative extent of surface mining disturbance between 1985–2005 across the study area (left). The amount of disturbance estimated within each county was regressed against cumulative county-level coal production reported for this period (right). Although Pike County, KY had more than twice the mining disturbance compared to any other county, confidence intervals around the slope estimates with the Pike County data included (95% CI = 8,320–11,300) overlapped with the confidence interval estimates for the slope value when this data point was omitted (95% CI = 9,650–13,340; confidence interval estimates calculated using the *confint* function in CRAN-R statistical software). Nonetheless, given this single data point exerts high leverage on the regression results, we use the values for the analysis in which this data point is excluded.

We found a surprisingly strong relationship where for every hectare of disturbance 11,500 short tons of coal are produced (1 short ton  = 907.2 kg; y = 11,500×−6.23×10^6^, R^2^  = 0.79) or, inversely, for each ton of coal 0.87 m^2^ of landscape is disturbed (fig. 1). Bituminous coal from the Central Appalachians has an average density of 1.32 g cm^−3^ [22]. If 11,500 tons of coal are produced per hectare disturbed, this would translate to an average coal seam thickness of only ∼1 m, compared to reported measurements of coal seam thicknesses for this area of ∼1.5 m [23]. However, our aerial estimates of disturbance include both the excavation of material overlying coal seams, as well as areas where mining spoil is displaced. Thus, our analysis suggests that the total footprint of MTR disturbances may exceed the spatial extent of underlying coal deposits by a factor of 1.5.

Previous assessments of stream impairment indicated that, on average, 27.0–30.2 m of stream impairment occurs for every hectare of surface mining in the Central Appalachians (Table 1). Given 11,500 tons of coal are produced per hectare disturbed, we estimate 0.25 cm of stream length was impaired, on average, for every ton of coal produced (Table 1).

Previous studies also indicate that mined sites reclaimed to forests and mined sites reclaimed to grasslands had C sequestration rates that were 32 and 222 g C m^−2^ yr^−1^ lower, respectively, compared to unmined forest ecosystems (Table 2). Despite this large range in potential foregone ecosystem C sequestration resulting from mining, the vast majority of reclaimed mines are seeded with grasses and support little woody vegetation regrowth even many years after site reclamation [2]. Because of this, we expect most mined ecosystems to exhibit values towards the upper end of this range. Based on our regression slope of 11,500 ton coal ha^−1^ and using the forgone C sequestration value of 222 g C m^−2^ yr^−1^, every ton of coal produced from the Central Appalachians is estimated to result in 193 g C ton coal^−1^ yr^−1^ of lost ecosystem C sequestration potential.

The average bituminous coal from this region has a carbon content of 80% [24]. Given 11,500 tons of coal are harvested for every hectare, ∼0.83 Mg C m^−2^ is released as CO_2_ through combustion assuming 99% of the C in coal is converted to CO_2_ [25]. Based on the C sequestration potential of these ecosystems it would take ∼5,000 years for any given hectare of former mines reclaimed to grassland to sequester the carbon released from combustion of the coal removed from that hectare, assuming these ecosystems could persist in an accumulating stage over these time periods. For those rare surface mines where forest regrowth is achieved, it would still take ∼2,150 years for these forested hectares to accumulate sufficient C in soils and tree biomass to sequester what was emitted through coal combustion.

**Figure 2:**
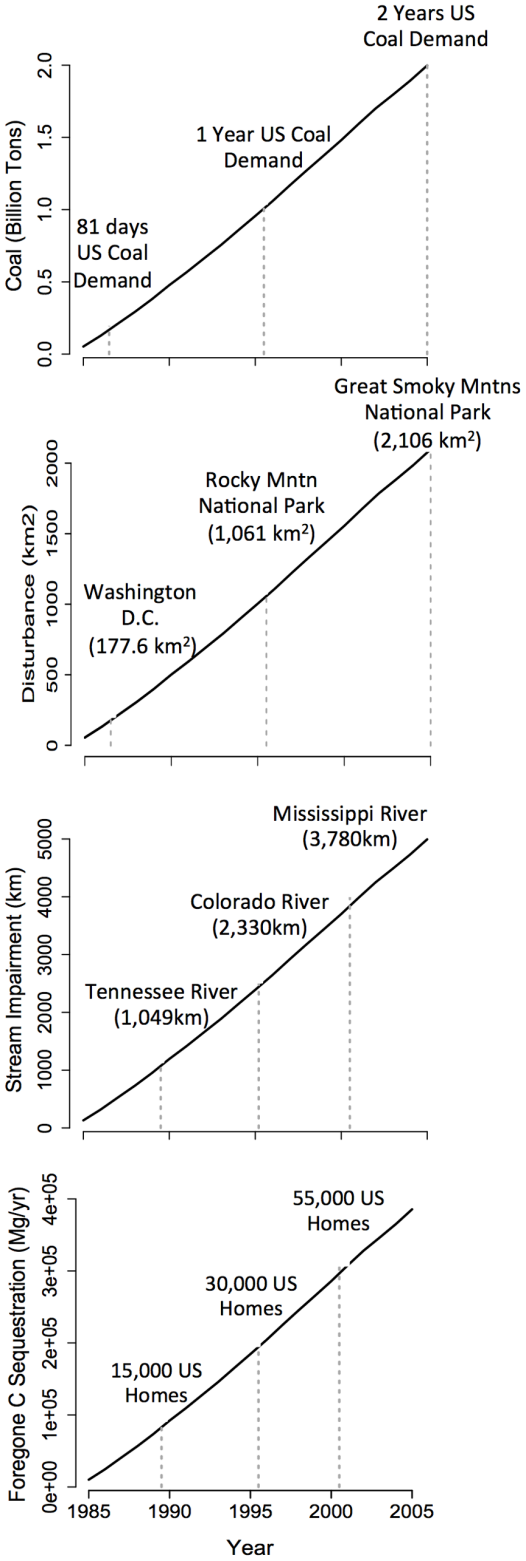
Scaling the environmental costs of coal production. Cumulative surface coal production across the 47 county study area is shown for the period 1985–2005 (A). Cumulative surface mining disturbance (B), stream length impairment (C), and forgone C sequestration (D) were estimated by multiplying the respective per-unit-coal values by the cumulative coal production data. Vertical dashed lines indicate comparative values for contextualization (*see* Table S2 in File S1).

While 0.25 cm of stream channel impairment or 193 g yr^−1^ of lost C sequestration per ton of coal may not sound like alarming environmental costs, it is difficult to appreciate these numbers unless they are placed in the context of regional coal production rates. Cumulative coal production within the study area totaled 1.93 billion tons over the 1985–2005 period (fig. 2A), or approximately two years worth of current US coal demand (Table S2 in File S1). To access this coal, nearly 2,000 km^2^ of land area was mined, which is comparable in size to the Great Smoky Mountains National Park (2,106 km^2^; fig. 2B). If all current US coal demand were to be supplied from surface mines in the Central Appalachians, an area equal in size to Washington DC (177.6 km^2^) would need to be mined every 81 days. To supply one year of current US coal demand would require converting 803 km^2^ of Appalachian mountains to surface mines, leading the to the biological impairment of an estimated ∼2,300 km of streams and forgone ecosystem C sequestration of ∼185,000 Mg C yr^−1^ (equivalent to the global warming potential of approximately 33,600 average US single family homes) (fig. 2C&D).

## Conclusions

Surface coal mining in the Central Appalachians has become an extremely contentious method for producing energy. While the scientific community has adequately demonstrated the *severity* of surface mining impacts [2], considerably less attention has been placed on understanding the *extent* of these environmental impacts and in providing the metrics necessary to compare these environmental costs to the obvious economic benefits of coal. This has been a critical shortcoming, as even the most severe impacts may be tolerable if they are believed to be sufficiently limited in extent. We show, however, that the extent of environmental impacts of these surface mining practices is staggering, particularly in terms of the amount of coal that is produced. Tremendous environmental capital is being spent to achieve what are only modest energy gains.

## Supporting Information

File S1 **Contains Table S1 and Table S2.**
(DOCX)

## References

[1] Saylor KL (2008) Land Cover Trends: Central Appalachians. Available: http://landcovertrends.usgs.gov/east/eco69Report.html. Accessed 20 February 2013.

[2] Palmer MA, Bernhardt ES, Schlesinger WH, Eshleman KN, Foufoula-Georgiou E, et al (2010) Mountaintop mining consequences. Science 327: 148–149. 20056876 10.1126/science.1180543

[3] EIA (2011) Coal Productivity by State and Mine Type, 2011, 2010. Available: http://www.eia.gov/coal/annual/pdf/table21.pdf. Accessed 20 February 2013.

[4] EIA (2011) Coal Prices, Selected Years, 1949–2011. Available: http://www.eia.gov/totalenergy/data/annual/pdf/sec7_21.pdf. Accessed 20 February 2013.

[5] WV State Tax Department (2007) West Virginia Coal Severance Tax Estimate. Available: http://www.state.wv.us/taxrev/uploads/sev400c.pdf. Accessed 20 February 2013.

[6] KY Office of State Budget (2010) Coal Severance and Processing Tax. Available: http://www.osbd.ky.gov/NR/rdonlyres/52EC9373-1916-401A-AB2F-3F3B52A5F20A/0/0810TEA_CoalServeranceandProcessingTax.pdf. Accessed 20 February 2013.

[7] GAO (2009) Surface Coal Mining: Characteristics of mining in Mountainous Areas of Kentucky and West Virginia. Available: http://www.gao.gov/new.items/d1021.pdf. Accessed: 20 February 2013.

[8] Simmons JA, Currie WS, Eshleman KN, Kuers K, Monteleone S, et al (2008) Forest to reclaimed mine land use change leads to altered ecosystem structure and function. Ecol Apps 18: 104–118. 10.1890/07-1117.1 18372559

[9] Amichev BY, Burger JA, Rodrigue JA (2008) Carbon sequestration by forests and soils on mined land in the Midwestern and Appalachian coalfields of the US. For Ecol & Manag 256: 1949–1959.

[10] Bernhardt ES, Lutz BD, King RS, Fay JP, Carter CE, et al (2012) How many mountains can we mine? Assessing the regional degradation of Central Appalachian rivers by surface coal mining. ES&T 46: 8115–8122. 10.1021/es301144q 22788537

[11] DOE (1980) Land use and energy. Available: http://www.osti.gov/energycitations/servlets/purl/6300166-L4I340/6300166.pdf. Accessed 20 February 2013.

[12] Pouch GW, Campagna DJ (1990) Hyperspherical direction cosine transformation for separation of spectral illumination information in digital scanner data. Photogrammetric Engineering and Remote Sensing 56: 475–479.

[13] Anderson JR, Hardy EE, Roach JT, Witmer RE (1976) A land use and land cover classification system for use with remote sensor data. US Geological Survey Professional Paper 964. Available: http://landcover.usgs.gov/pdf/anderson.pdf. Accessed 20 February 2013.

[14] Short NM (1982) The remote sensing tutorial. NASA. Available: http://rst.gsfc.nasa.gov. Accessed 20 February 2013.

[15] Kentucky Geological Survey (2013) Coal Production Database. Available: http://www.uky.edu/KGS/coal/production/kycoal01.htm. Accessed 20 February 2013.

[16] West Virginia Geological and Economic Survey (2010) Coal Summary Statistics. Available: http://www.wvgs.wvnet.edu/www/datastat/coalsummary/coal_summary.asp. Accessed 20 February 2013.

[17] R Development Core Team (2010) Available: http://www.R-project.org.Accessed 20 February 2013.

[18] Barbour MT, Gerritsen J, Snyder BD, Stribling JB (1999) In Rapid Bioassessment Protocols for Use in Streams and Wadeable Rivers: Periphyton, Benthic Macroinvertebrates and Fish, Second ed.; EPA 841 – B-99–002. Available: http://water.epa.gov/scitech/monitoring/rsl/bioassessment/index.cfm. Accessed 20 February 2013.

[19] Pond GJ, Bailey JE, Lowman BM, Whitman MJ (2013) Calibration and validation of a regionally and seasonally stratified macroinvertebrate index for West Virginia wadeable streams. Environmental Monitoring and Assessment. 185: 1515–1540. 10.1007/s10661-012-2648-3 22580746

[20] Baker ME, King RS (2010) A new method for detecting and interpreting biodiversity and ecological community thresholds. Methods Ecol. Evol. 1(1): 25–37.

[21] Zipper CE, Burger JA, Skousen JG, Angel PN, Barton CD, et al (2011) Restoring forests and associated ecosystem services on Appalachian coal surface mines. Environmental Management 47: 751–765. 21479921 10.1007/s00267-011-9670-z

[22] Wood GH, Kehn TM, Carter MD, Culbertson WC (1980) Coal Resource Classification System of the US Geological Survey. Available: http://pubs.usgs.gov/circ/c891/table2.htm. Accessed 20 February 2013.

[23] EPA (2003) Affected Environment and Consequences of MTM/VF. Available: http://www.epa.gov/region03/mtntop/pdf/III_affected-envt-consequences.pdf. Accessed 20 February 2013.

[24] USGS (2009) Coal Resource Availability, Recoverability, and Economic Evaluations in the United States – A Summary. Available: http://pubs.usgs.gov/pp/1625f/downloads/ChapterD.pdf. Accessed 20 February 2013.

[25] EPA (1998) Bituminous and subbituminous coal combustion. Available: http://www.epa.gov/ttnchie1/ap42/ch01/final/c01s01.pdf. Accessed 20 February 2013.

