# Supplementary material for: The Environmental Price Tag on a Ton of Mountaintop Removal Coal
Source: PLoS One. 2013 Sep 11;8(9):e73203. doi: 10.1371/journal.pone.0073203 (PMC3770658; doi:10.1371/journal.pone.0073203)
Supplement: File S1 — Contains Table S1 and Table S2. (DOCX) [file pone.0073203.s001.docx]

**SUPPORTING INFORMATION**

**Table S1. Historical County Coal Production Data.**

| **County** | **State** | **County Area (Ha)** | **1985-2005 Mining Disturbance (Ha)** | **Percent of County Area Mined 1985-2005** | **1985-2005 Surface Mining Coal Produced (tons)^a^** |
| --- | --- | --- | --- | --- | --- |
| Bath | KY | 73,543.6 | 0.0 | 0.0% | 0 |
| Bell | KY | 97,019.0 | 6,002.0 | 6.2% | 42,182,364 |
| Boyd | KY | 41,563.5 | 823.6 | 2.0% | 1,591,743 |
| Breathitt | KY | 128,814.0 | 9,094.5 | 7.1% | 91,799,271 |
| Carter | KY | 106,343.8 | 1,294.6 | 1.2% | 873,961 |
| Clay | KY | 122,763.1 | 2,238.6 | 1.8% | 9,272,446 |
| Elliott | KY | 59,632.0 | 828.0 | 1.4% | 1,588,168 |
| Estill | KY | 66,571.5 | 0.2 | 0.0% | 77,682 |
| Floyd | KY | 103,071.4 | 7,745.9 | 7.5% | 63,624,700 |
| Greenup | KY | 88,660.0 | 1,784.5 | 2.0% | 6,845,753 |
| Harlan | KY | 125,953.8 | 6,531.9 | 5.2% | 38,619,102 |
| Jackson | KY | 89,564.8 | 207.6 | 0.2% | 1,715,724 |
| Johnson | KY | 68,058.0 | 2,142.6 | 3.1% | 6,474,616 |
| Knott | KY | 89,774.0 | 10,580.9 | 11.8% | 85,117,875 |
| Knox | KY | 99,847.0 | 3,050.4 | 3.1% | 7,881,011 |
| Laurel | KY | 115,883.4 | 539.3 | 0.5% | 2,006,439 |
| Lawrence | KY | 108,168.7 | 3,018.0 | 2.8% | 7,926,500 |
| Lee | KY | 55,123.3 | 457.7 | 0.8% | 1,557,600 |
| Leslie | KY | 103,248.8 | 6189.3 | 6.0% | 35,807,096 |
| Letcher | KY | 90,576.1 | 11,191.8 | 12.4% | 72,453,414 |
| Magoffin | KY | 79,330.2 | 3,689.6 | 4.7% | 19,045,384 |
| Martin | KY | 60,065.8 | 9,904.2 | 16.5% | 107,546,148 |
| McCreary | KY | 111,320.0 | 167.2 | 0.2% | 367,438 |
| Menifee | KY | 54,791.4 | 0.0 | 0.0% | 0 |
| Montgomery | KY | 52,019.4 | 0.0 | 0.0% | 0 |
| Morgan | KY | 102,547.7 | 969.2 | 0.9% | 986,540 |
| Owsley | KY | 50,839.1 | 1,115.7 | 2.2% | 4,047,685 |
| Perry | KY | 89,374.6 | 13,516.5 | 15.1% | 154,097,558 |
| Pike | KY | 209,478.2 | 27,215.2 | 13.0% | 216,287,169 |
| Powell | KY | 47,770.0 | 0.0 | 0.0% | 0 |
| Rowan | KY | 76,125.0 | 0.0 | 0.0% | 0 |
| Whitley | KY | 114,908.2 | 4,890.7 | 4.3% | 13,142,370 |
| Wolfe | KY | 58,016.3 | 1,170.5 | 2.0% | 3,620,772 |
| Boone | WV | 132,019.6 | 12,835.7 | 9.7% | 177,795,332 |
| Clay | WV | 89,698.9 | 2,252.3 | 2.5% | 60,743,790 |
| Fayette | WV | 176,818.3 | 3,818.8 | 2.2% | 46,362,732 |
| Kanawha | WV | 234,480.3 | 4,572.9 | 2.0% | 122,987,677 |
| Lincoln | WV | 112,386.3 | 1,909.9 | 1.7% | 8,519,631 |
| Logan | WV | 116,433.7 | 9,796.1 | 8.4% | 183,425,300 |
| McDowell | WV | 136,333.4 | 2,913.8 | 2.1% | 23,517,204 |
| Mercer | WV | 110,112.5 | 316.3 | 0.3% | 337,796 |
| Mingo | WV | 112,394.6 | 10,141.9 | 9.0% | 132,771,904 |
| Nicholas | WV | 167,569.0 | 6,750.5 | 4.0% | 76,559,886 |
| Raleigh | WV | 157,189.1 | 1,810.0 | 1.2% | 12,919,464 |
| Wayne | WV | 136,037.7 | 1,282.1 | 0.9% | 14,682,591 |
| Webster | WV | 144,799.1 | 3,210.4 | 2.2% | 50,027,526 |
| Wyoming | WV | 131,849.9 | 3,600.6 | 2.7% | 27,143,738 |
| **TOTALS:** |  | **4,898,888.2** | **201,571.3** | **3.9% (average)** | **1,934,351,100** |

^a^ 1985 data for WV were not available. However, 1986 values were duplicated to approximate the missing 1985 values. Annual surface mining coal production increased by nearly 230% from 1986 (26.7 million tons yr^-1^) to 2005 (60.9 million tons yr^-1^) from these 14 counties, and the sum of coal produced across counties in 1986 accounted for only 2.7% of the cumulative coal produced from all counties between 1986-2005. As a result, any amount of innaccuracy introduced from using 1986 values to approximate those for 1985 has negligible effect on our analysis.

**Table S2: Scaling Costs and Benefits.**

| **Comparison** | **Panel in Figure 2** | **Data Source and Calculation** |
| --- | --- | --- |
| *Cumulative coal production* |  | 1. Cumulative coal production data in Panel A are derived from state production records (*see* Table S1 for data sources). |
| *vs.* | A |  |
| *Current US demand* |  | 1. 0.92 billion tons of bituminous coal from Central Appalachia provides one-year of US coal energy demand*^a^* |
| *Cumulative disturbance* |  | 1. Estimated by multiplying the per unit coal value (0.87m^2^/ ton coal) by cumulative coal produced (fig 2, Panel B). |
| *vs.* | B |  |
| *Prominent Geographic Features* |  | 1. Sources for areas of geographic features: Washington D.C. (177.6km^2^), Rocky Mountain National Park (1,601km^2^), Great Smoky Mountains National Park (2,106km^2^)^b^ |
| *Cumulative stream impairment* |  | 1. Estimated by multiplying the per unit coal value (0.25 cm / ton coal) by cumulative coal produced (fig 2, Panel C). |
| *vs.* | C |  |
| *Major US Rivers* |  | 1. Sources for lengths of major US rivers: Tennessee River (1,049 km), Colorado River (2,330 km), Mississippi River (3,780km)^b^ |
| *Foregone C Sequestration* |  | 1. Estimated by multiplying the per unit coal value (193 gC / ton coal / yr) by cumulative coal produced (fig 2, Panel D). |
| *vs.* | D |  |
| *C Footprint of US Homes* |  | 8. The average C footprint of a US home is reported by US EPA as 19.43 metric tons CO_2_ yr^-1[c]^. |

^a^ We represent the scaled environmental costs in the context of current annual US coal demand (2011). Different grades of coal, however, can range widely in heat content, and the bituminous coal primarily found throughout the Appalachians has an average heat content of 24 million Btu per ton; subbituminous coal, the other major form of coal produced in the US, has an average heat content of only 18 million Btu per ton (Btu = British thermal unit) [25]. In 2011, ~1.1 billion short tons of coal were produced in the US [26], yielding an estimated 22.18 quadrillion Btu [27]. If this same amount of coal energy were produced exclusively from the higher energy content bituminous coal common to the Appalachians, only 0.92 billion short tons would result in the same amount of total energy.

^b^ see ref 28

^c^ see ref 29

**SUPPLEMENTARY REFERENCES**

1. DOE (1995) Coal Data: A Reference. Available: ftp://ftp.eia.doe.gov/coal/006493.pdf. Accessed 20 February 2013.
2. EIA (2011) Annual Coal Production Data 2011. Available: http://www.eia.gov/coal/annual/pdf/table1.pdf. Accessed 20 February 2013.
3. EIA (2011) Primary Energy Production by Source, Selected Years, 1949-2011. Available: http://www.eia.gov/totalenergy/data/annual/pdf/sec1_7.pdf. Accessed 20 February 2013.
4. Merriam Webster Online Encyclopedia, <http://www.merriam-webster.com>.
5. EPA (2013) Clean Energy Resources. Available: http://www.epa.gov/cleanenergy/energy-resources/refs.html. Accessed 20 February 2013.
